# Global Spread of Human Chromoblastomycosis Is Driven by Recombinant *Cladophialophora carrionii* and Predominantly Clonal *Fonsecaea* Species

**DOI:** 10.1371/journal.pntd.0004004

**Published:** 2015-10-23

**Authors:** Shuwen Deng, Clement K. M. Tsui, A. H. G. Gerrits van den Ende, Liyue Yang, Mohammad Javad Najafzadeh, Hamid Badali, Ruoyu Li, Ferry Hagen, Jacques F. Meis, Jiufeng Sun, Somayeh Dolatabadi, Bernard Papierok, Weihua Pan, G. S. de Hoog, Wanqing Liao

**Affiliations:** 1 Shanghai Institute of Medical Mycology, Changzheng Hospital, Shanghai, China; 2 First Hospital of Xinjiang Medical University, Xinjiang, China; 3 CBS-KNAW Fungal Biodiversity Centre, Utrecht, The Netherlands; 4 British Columbia Public Health Microbiology & Reference Laboratory at BCCDC site, Provincial Health Services Authority, Vancouver, Canada; 5 Department of Parasitology and Mycology & Cancer Molecular Pathology Research Center, Ghaem Hospital, School of Medicine, Mashhad University of Medical Sciences, Mashhad, Iran; 6 Department of Medical Mycology and Parasitology / Invasive Fungi Research Center (IFRC), School of Medicine, Mazandaran University of Medical Sciences, Sari, Iran; 7 Research Center for Medical Mycology, Beijing Medical University, Beijing, China; 8 Department of Medical Microbiology and Infectious Diseases, Canisius Wilhelmina Hospital, Nijmegen, The Netherlands; 9 Radboud University Medical Center, Nijmegen, The Netherlands; 10 Guangdong Provincial Institute of Public Health, Guangdong Provincial Center for Disease Control and Prevention, Guangzhou, China; 11 Cellular and Molecular Research Center, Sabzevar University of Medical Sciences, Sabzevar, Iran; 12 Institute Pasteur, Paris, France; 13 King Abdulaziz University, Jeddah, Saudi Arabia; University of Tennessee, UNITED STATES

## Abstract

Global distribution patterns of *Cladophialophora carrionii*, agent of human chromoblastomycosis in arid climates of Africa, Asia, Australia, Central-and South-America, were compared with similar data of the vicarious *Fonsecaea* spp., agents of the disease in tropical rain forests. Population diversities among 73 *C*. *carrionii* strains and 60 strains of three *Fonsecaea* species were analyzed for rDNA ITS, partial β-tubulin, and amplified fragment-length polymorphism (AFLP) fingerprints. Populations differed significantly between continents. Lowest haplotype diversity was found in South American populations, while African strains were the most diverse. Gene flow was noted between the African population and all other continents. The general pattern of *Fonsecaea* agents of chromoblastomycosis differed significantly from that of *C*. *carrionii* and revealed deeper divergence among three differentiated species with smaller numbers of haplotypes, indicating a longer evolutionary history.

## Author Summary

Chromoblastomycosis is a severely mutilating disease caused commonly by two fungal species groups in *Cladophialophora* and *Fonsecaea*, which occur in arid climates or in the tropical rain forest, respectively. Understanding the population structure and evolution of chromoblastomycosis is important to reconstruct the epidemiological history and to identify evolutionary process and environmental drivers of disease spread. We used two sequence markers; internal transcribed spacer (ITS) region of rDNA and the partial ß-tubulin (*BT2*) gene, as well as AFLP markers, respectively, to investigate the population structure and differentiation of 73 *C*. *carrionii* strains and 60 strains of three *Fonsecaea* species. These agents of human disease have diverse distribution patterns and population dynamics even though they share a common ancestor within the order *Chaetothyriales*. It may be speculated that they have taken advantage of and adapted to human hosts as a new ecological niche.

## Introduction

Chromoblastomycosis is a serious fungal skin disease that may lead to severe morbidity The disease is characterized histologically by muriform cells that cause chronic inflammation of the stratum spinosum or stratum corneum, as well as infection of subcutaneous tissues, Infection may lead to cauliflower-like eruptions on the skin, hyperkeratosis, or intermediate forms, depending on the type of interaction between host and fungal cells [1]. The disease is seen worldwide, particularly in tropical and subtropical regions with higher prevalence in Southern Africa and Madagascar [2,3], Latin America (Mexico, Brazil, Venezuela){Queiroz-Telles, 2013 #2613}[1,4,5], East Asia and Australia [6,7]. In hyperendemic regions such as arid parts of Venezuela, Yegres [8] and Yegüez-Rodriguez [9] noted a frequency of 16 cases/1,000 persons. Although several fungal species are potential etiologic agents of the disease, *Fonsecaea pedrosoi* and *Cladophialophora carrionii* are prevalent in the endemic areas.

The disease is difficult to treat for clinicians due to its recalcitrant nature, which may lead to severe clinical forms with high morbidity. Success of treatment is related to the identity of the causative agent, the clinical form and severity of the chromoblastomycosis lesions.

A limited number of fungi has been reported as the etiological agents. *Cladophialophora carrionii* is the predominant agent of the disease under arid, desert-like climatic conditions [10]. *Cladophialophora samoensis* was reported as a single case from humid tropical Samoa in Polynesia [11]. Members of *Fonsecaea* are responsible for the diseases in the subtropical rain forest and adjacent regions [12–14]. In addition, *Rhinocladiella aquaspersa* was found in various climate zones [15,16]. Sporadic cases of chromoblastomycosis-like infections have been reported by *Phialophora verrucosa*, *Exophiala spinifera* and *Exophiala dermatitidis* [17–19]. All the above genera belong to the same family *Herpotrichiellaceae* in the fungal order *Chaetothyriales* [20].

Chromoblastomycosis is not contagious and is probably acquired by traumatic inoculation of contaminated material such as plant thorns. The agents of chromoblastomycosis must be environmental, but apparently have higher degrees of adaptation to the human host than their strictly saprobic siblings. Perhaps mammals serve as a reservoir for the pathogenic species, as is suggested by selective isolation methods of these fungi using mouse baits which were successful in recovering the clinical species [21]. So far no known animal vector has been reported, but we cannot rule out the possibility that human or other mammal hosts can play a role in the evolution and dispersal of the etiologic agents.

The distribution of the etiologic agents is influenced by environmental factors prevailing in their habitats. However, when environmental samples are used for isolation of black fungi, mostly species that are closely related, strictly saprobic siblings are yielded, and only exceptionally any of the potential agents of disease [22]. De Hoog et al. [10] showed that all clinical strains from an arid climate zone in Venezuela were *Cladophialophora carrionii*, while morphologically identical strains from cactus thorns in the same region concerned a related, but different *Cladophialophora* species. Similar findings were reported with *Fonsecaea* [23] in the tropical rain forest.

Despite the global occurrence of chromoblastomycosis, the population structure of the etiological agents is not well investigated. Understanding the population structure and evolution of chromoblastomycosis is important to reconstruct epidemiological history and to identify evolutionary process and environmental drivers of disease spread. The main objective of the present study was to determine the genetic diversity and spatial pattern of *Cladophialophora* and *Fonsecaea* agents of chromoblastomycosis isolated globally from clinical and environmental samples. We used two sequence markers; internal transcribed spacer (ITS) region of rDNA and the partial ß-tubulin (*BT2*) gene, as well as AFLP markers, respectively, to investigate the population structure and differentiation of 73 *C*. *carrionii* strains and 60 strains of three *Fonsecaea* species. Specifically we were interested in: i) comparing the population structure of these major fungal pathogens that are the prevalent agents of chromoblastomycosis, and ii) deducing the potential source and pattern of the endemism and whether multiple introductions of the pathogen have occurred.

## Results

### Phylogeny of Genera Belonging to the Order *Chaetothyriales*

A representative overview of the *Chaetothyriales* (family *Herpotrichiellacae*) was constructed based on ITS sequence data of 81 strains using neighbor joining with Kimura 2—parameter and gamma correction substitution model, 1000 bootstrap replications and *Knufia epidermidis* as outgroup (S1 Fig). The prevalent agents of chromoblastomycosis in *Cladophialophora* and *Fonsecaea* covering the great majority of published cases formed two separate clusters. The rare species *Rhinocladiella aquaspersa* formed a separate clade. Species where occasional strains in *Exophiala* and *Phialophora* causing chromoblastomycosis have been reported are all located in separate clades (dotted lines in S1 Fig).

### Population Genetics

The total length of concatenated ITS and *BT2* sequences (excluding gaps/missing data) in *Cladophialophora carrionii* and *Fonsecaea* species were 854 bp and 759 bp, respectively; diversities are shown in Table 1.

**Table 1:** Diversity of ITS, *BT2* and concatenated ITS-*BT2* datasets in C*ladophialophora carrionii* and *Fonsecaea* spp.

Strains (N)				Polymorphic site (*S*)			Haplotype diversity (*H*_d)_			Nucleotide diversity (π)		
	Singletons			Parsimonious informative								
	ITS	*BT2*	ITS-*BT2*	ITS	*BT2*	ITS-*BT2*	ITS (n)	*BT2* (n)	ITS-*BT2* (n)	ITS	*BT2*	ITS-*BT2*
***C*. *carrionii* (67)**	11	13	24	3	16	19	0.4969 (10)	0.7437 (20)	0.8480 (35)	0.00675	0.00877	0.00621
	1	7	8	20	20	40	0.7989 (11)	0.7732 (13)	0.8718 (18)	0.01386	0.01823	0.01595

(N) = strains number, (n) = number of haplotypes

In *C*. *carrionii*, 14 polymorphic sites (*S*) in ITS consisted of 11 singletons and 3 parsimonious informative sites. Gene fragment *BT2* had 29 polymorphic sites (*S*) of which 13 were singletons and 16 were parsimonious informative sites. Haplotype diversity (*H*_*d*_) for ITS (n = 10) was 0.4969, and for *BT2* (n = 20) 0.7437; nucleotide diversity (π) for ITS was 0.00675 and for *BT2* 0.00877. In the concatenated dataset (ITS-*BT2*), 43 polymorphic sites (*S*) consisted of 24 singletons and 19 parsimonious informative sites. Haplotype diversity (*H*_*d*_) (n = 35, S2 Fig) was 0.8480 with a nucleotide diversity (π) of 0.00621.

In *Fonsecaea* species, 21 polymorphic sites (*S*) in ITS consisted of 1 singleton and 20 parsimonious informative sites. Gene fragment *BT2* showed 7 singletons and 20 parsimonious informative sites; haplotype diversity (*H*_*d*_) of ITS (n = 11) was 0.7989, and of *BT2* (n = 13) 0.7732. In the ITS alignment, 4 unique mutations were found in *F*. *nubica*, 1 in *F*. *monophora*, and 6 in *F*. *pedrosoi*. The *BT2* alignment showed 2 unique mutations in *F*. *nubica*, 3 in *F*. *monophora*, and 5 in *F*. *pedrosoi* (S2 Fig). The nucleotide diversity (π) in *Fonsecaea* species was higher than that of *C*. *carrionii*, namely 0.01386 for ITS and 0.01823 for *BT2*. In the concatenated dataset (ITS-*BT2*) (n = 18, S2 Fig), 48 polymorphic sites (*S*) consisted of 8 singletons and 40 parsimonious informative sites. Haplotype diversity (*H*_*d*_) (n = 18) was 0.8718 and nucleotide diversity (π) was 0.01595.

**Fig 1:**
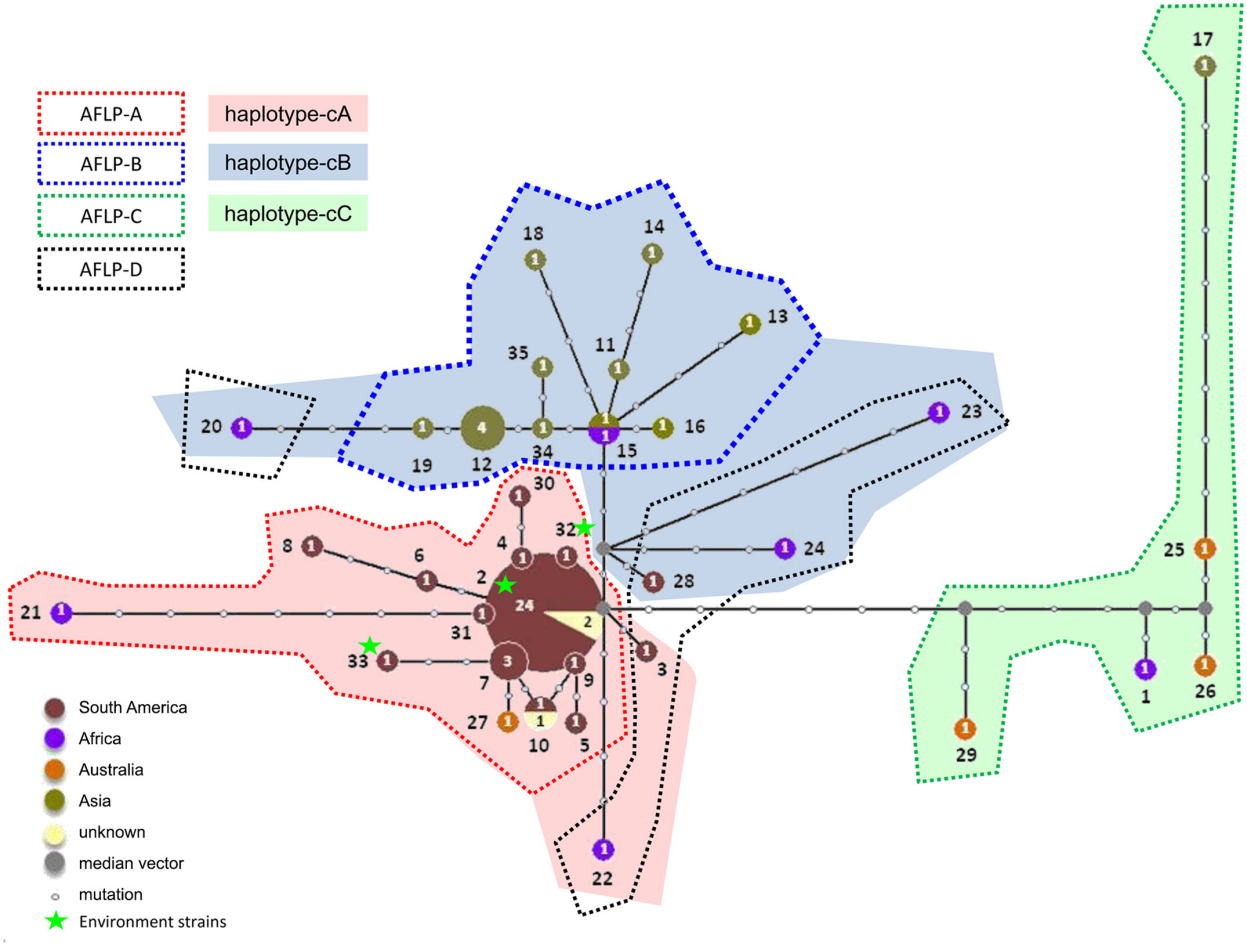
*Cladophialophora carrionii* ITS-*BT2* haplotype network, with geographical distribution, gaps and missing data excluded. Three main clusters (cA~cC) were recognizable in concatenated ITS-*BT2* sequence data of *C*. *carrionii*. Cluster cC took a most distant position and harbored the largest diversity. Cluster cB was dominated by Asian strains. Strains of group cA nearly all came from South or Central America. These groups matched with groups (AFLP-A~AFLP-D) given by AFLP analysis. The majority of African strains clustered close to group AFLP-A, and subcluster AFLP-D which contain the majority of strains clustered in sequence-based group cA’ and cB’.

Haplotype network analysis of concatenated ITS-*BT2* sequence data of *Cladophialophora carrionii* revealed three main clusters (cA~cC) largely correlated with the geographic locations with potential recombination event (Figs 1 and S2). Cluster cC was deeply diverged from the other two clusters (cA and cB). Several cC strains originated from Australia, while haplotype 1 originated from Madagascar, Africa; haplotype 17 from Japan deviated strongly from Australian strains. Cluster cB was dominated by Asian strains (haplotype 12 was common). A group paraphyletic to cB (cB’) contained haplotypes mainly from Africa. Haplotype 10 was a recombinant. Strains of group cA nearly all came from South or Central America; haplotype 27 (1 strain) originated from Australia, and a deep diverging haplotype 21 (1 strain) came from Africa. Haplotype 2, the most frequent in S. America (61%, 24 haplotype 2 in total 39 haplotype of S. America), was not recorded in other continents except two strains from unknown source. The paraphyletically located haplotypes 3 (S. America) and 22 (Africa) were taken together under cA’.

**Fig 2:**
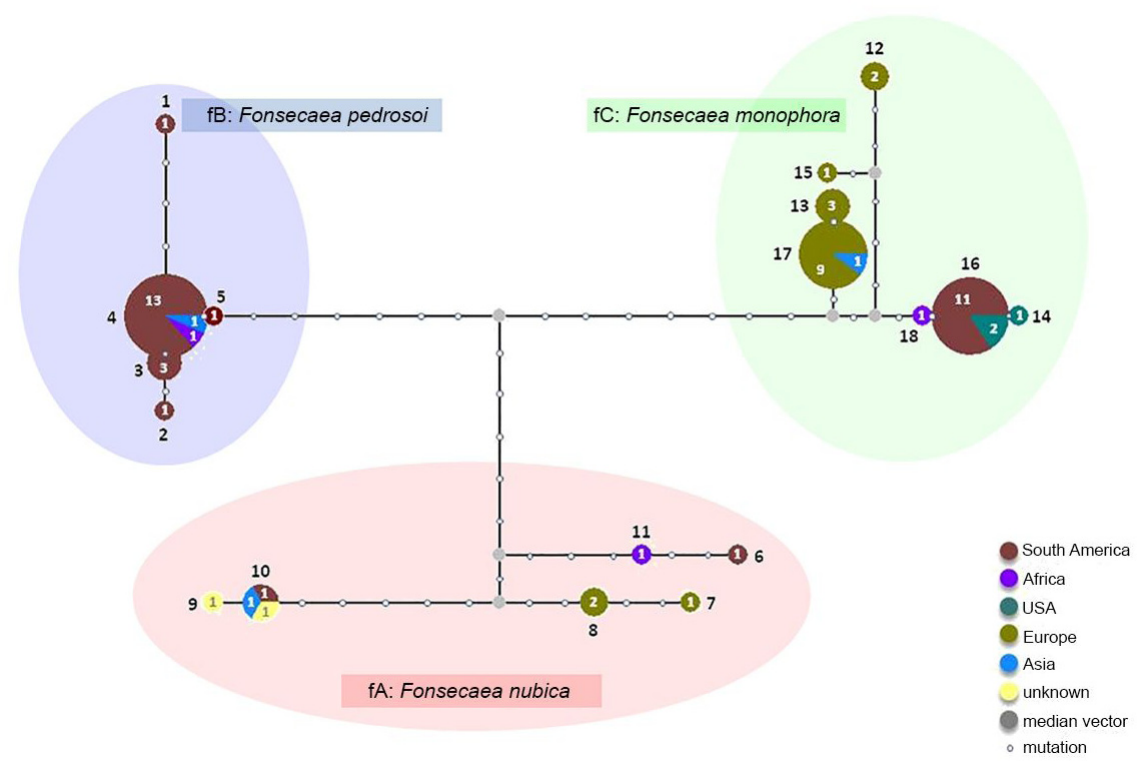
*Fonsecaea monophora*, *F*. *pedrosoi*, *F*. *nubica* ITS-*BT2* haplotype network, with geographical distribution, gaps and missing data excluded. In combined ITS-*BT2* sequence data of *Fonsecaea* spp., three widely different groups were recognizable (fA~fC), representing the three described *Fonsecaea* spp.

In the ITS-*BT2* haplotype network of *Fonsecaea* species (Fig 2), three deeply diverged groups (fA~fC) were recognizable, representing the three described *Fonsecaea* species, each with 5–7 haplotypes. *Fonsecaea nubica* had global distribution and the highest diversity, the minor haplotypes being geographically isolated from each other. *Fonsecaea pedrosoi* had five haplotypes, and its major haplotype (13 out of 15 samples) came from South America. *Fonsecaea monophora* was the main species in Asia and South America but was less common in Africa (Fig 2). The major haplotypes in Asia (hap 17) and S. America (hap 16) clustered geographically.

### AFLP Profiles of *Cladophialophora carrionii*

AFLP analysis with 73 strains of *C*. *carrionii* revealed four main groups marked as AFLP-A to AFLP-D (Figs 1 and S3), dominated by South American, Asian, and Australian strains, respectively. The main groups (AFLP-A~AFLP-C) matched with groups (cA~cC) (Fig 1) as found on the basis of sequence data. The majority of South American strains clustered close to group AFLP-A, but composing the approximate subcluster AFLP-D, containing the majority of strains in sequence-based group cA’ and cB’ (Fig 1). The environmental strains from cactus debris in Venezuela (CBS 861.96, CBS 862.96, CBS 863.96) clustered in AFLP-A and in sequence group cA (Figs 1 and S3). AFLP data for *Fonsecaea* species with species-specific degrees of diversity matching the above sequence data were discussed by [24] who used the same strains of *Fonsecaea* species as the ones studied here.

### Neutrality Test Based on Concatenated ITS-*BT2* Data

To observe evidence of deviation from neutrality, Tajima’s *D* was calculated from DnaSP 5.1. The sequence data were statistically significant (*P* < 0.05) deviating from neutrality with a Tajima’s *D* value of −2.0147. Fu & Li’s D* and F* test also showed negative values: −3.3596 and −3.3882, respectively (*P* < 0.02) indicating an excess of low-frequency mutations, most likely, due to population expansion (Table 2). This was confirmed by the amount of singletons (34; Table 1) which was more than 50% of the total amount of variable sites.

**Table 2:** Neutrality test of *Cladophialophora carrionii* and *Fonsecaea* spp. based on concatenated ITS-*BT2* data.

Strains (n)	Tajima’s D	Fu & Li’s D*	Fu & Li’s F*
***Cladophialophora carrionii* (n = 67)**	-2.0147 (p< 0.05)	-3.3596 (p < 0.05)	-3.3882 (p < 0.02)
***Fonsecaea spp*. (n = 60)**	0.4395 (p > 0.10)	0.5412 (p > 0.10)	0.5995 (p > 0.10)

D* Excess of low-frequency variants, as a result from population expansion, weak negative selection or positive selection

F* Excess of intermediate-frequency alleles, as a result from population bottlenecks, structure and/or balancing selection

Tajima’s *D* of *Fonsecaea* species was positive (0.4395), but was not statistically supported (*P >* 0.10). This was also the case for Fu & Li’s D* and F* tests (0.5412 and 0.5995; *P* > 0.10), showing some evidence of balancing selection (Table 2).

### Population Structure and Differentiation

**Fig 3:**
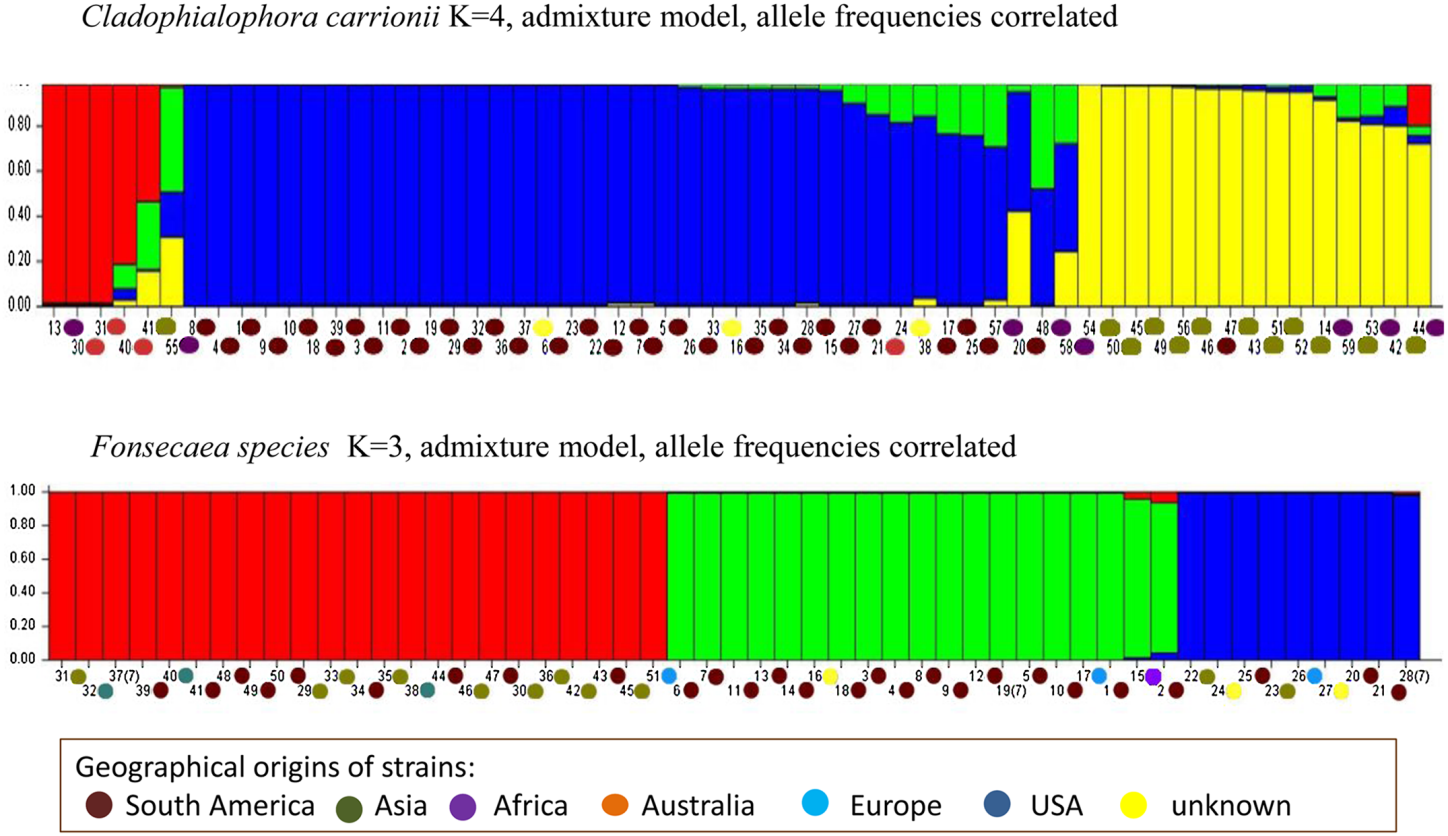
The population structure of *Cladophialophora carrionii* and *Fonsecaea* spp. with Q-hat order. Structure 2.3.4 used to establish the population structure of global isolates of *C*. *carrionii* isolates and *Fonsecaea* spp., *Cladophialophora carrionii* K = 4, admixture model, allele frequencies correlated; *Fonsecaea* species K = 3, admixture model, allele frequencies correlated. In the Structure display the color between parentheses depict the geographical origin of the haplotype group.

Structure analysis of haplotypes indicated Δ*K* = 4 in *C*. *carrionii* based on the Evanno method implemented in Structure Harvester (S4 Fig). Based on individual assignment, there were four genetic clusters that largely corresponded to the geographic locations of the samples. The clusters of Australia, Asia and South America are relatively homogeneous with high membership coefficients, in contrast to the cluster of African haplotypes which had a high level of admixture (Fig 3). Structure analyses of *Fonsecaea* haplotypes demonstrated Δ*K* = 3 (S4 Fig). The genetic clusters were related to the three described species as samples from the same genetic cluster were present in more than one geographic region (Fig 3).

In Table 3, population differentiations (*F*_*st*_ = 0.14974~0.54397) in *C*. *carrionii* were evidenced among subpopulations in Africa, Asia, and Central and South America. The least differentiated populations were observed between Africa and Australia, Africa and Asia, and Africa and South America, while the most differentiated populations were Asia and South America. Large population pair-wise *F*_*st*_ were also common across geographic groupings in *Fonsecaea* species, suggesting strong genetic differentiation increased with geographic distance (Table 4). The divergence among three described species was bigger than the geographic differentiation within each species, which was not reflected in the table. For instance, *F*. *monophora* was prevalent in Asia and S. America while *F*. *pedrosoi* was predominant in S. America only. The least differentiated population was found between Asia and Africa (*F*_*st*_ = 0.06351) where both *F*. *nubica* and *F*. *monophora* were both present. (Table 4).

**Table 3:** Genetic differentiation among populations occurring on different continents of *Cladophialophora carrionii* based on concatenated ITS-*BT2* sequence data, *Fst* indices for *Cladophialophora carrionii* (n = 67).

ITS-*BT2*	South America	Africa	Asia
**Africa**	0.17079	-	-
**Asia**	0.54397	0.15273	-
**Australia**	0.42917	0.14974	0.44305

*F*_*st*_ indices interpretion:

0–0.05: little genetic differentiation; 0.05–0.15: moderate genetic differentiation

0.15–0.25: great genetic differentiation; > 0.25: very great genetic differentiation (isolation)

**Table 4:** Genetic differentiation among populations occurring on different continents of *Fonsecaea* spp. based on concatenated ITS-*BT2* sequence, *Fst* indices for *Fonsecaea pedrosoi*, *F*. *monophora*, *F*. *nubica* (n = 60) groups.

ITS-*BT2*	South America	USA	Asia
**USA**	0.55487	-	-
**Asia**	0.35312	0.4766	-
**Africa**	n/c	0.31579	0.06351

*F*_*st*_ indices interpretion:

0–0.05: little genetic differentiation; 0.05–0.15: moderate genetic differentiation

0.15–0.25: great genetic differentiation; > 0.25: very great genetic differentiation (isolation)

### Recombination Analysis

**Fig 4:**
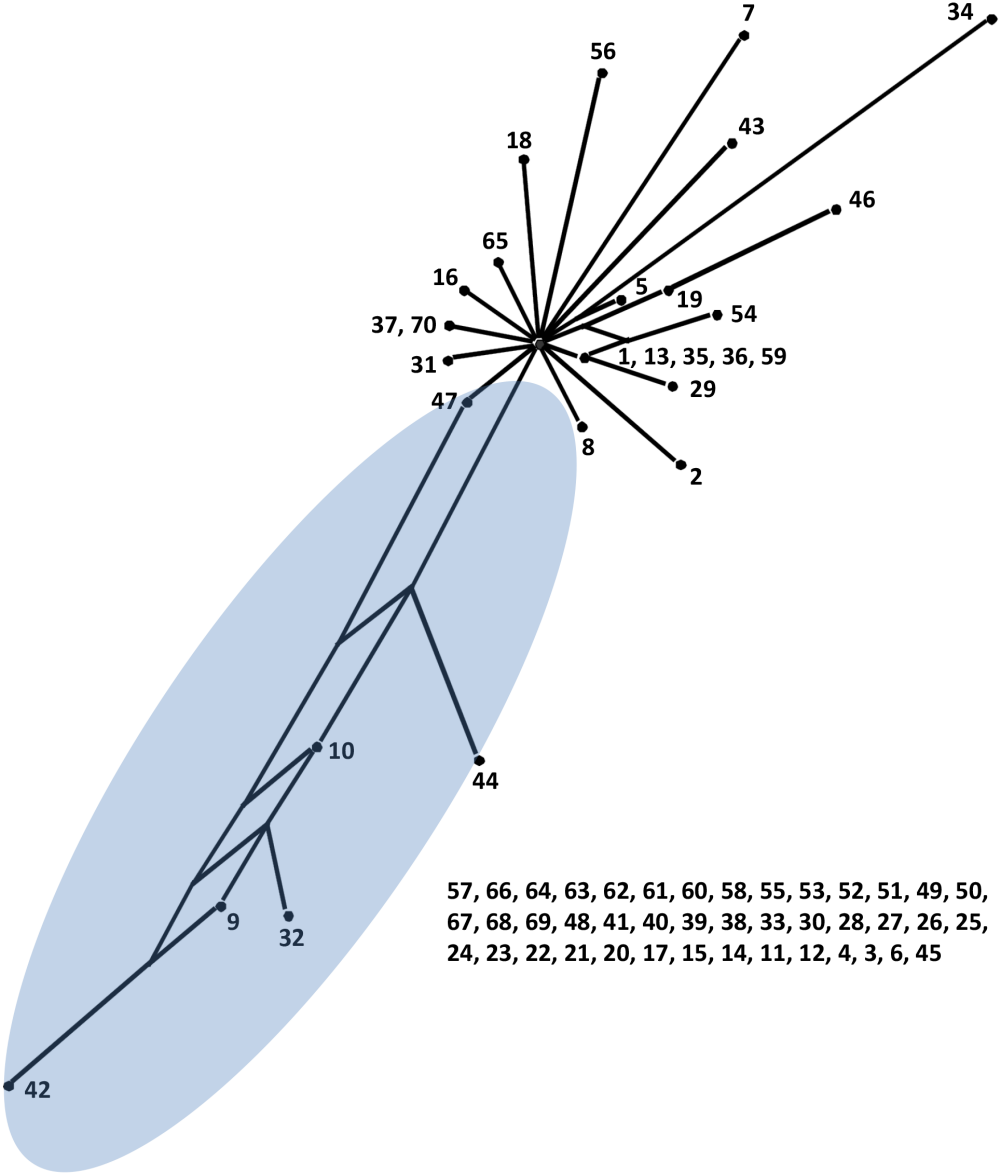
Split decomposition network of *Cladophialophora carrionii* constructed with Splitstree (S1 Text).

As shown in S3 Table, In *Cladophialophora carrionii*, Max χ^2^ detected statistically significant evidence for recombination in both datasets of *BT2* (*P* = 0.02) and ITS (*P* = 0.03), with the most significant breakpoint between strains CBS 117901 (Venezuela) and CBS 131854 (Madagascar). PHI showed statistically significant evidence for recombination, while Gard did not in *BT2* (PHI: P = 0.02; Gard negative). No recombination event was observed in ITS data using both PHI and Gard (PHI: P = 0.30; Gard negative). The split decomposition network (Fig 4, S1 Text) consisted of a prevalently star-like representation with a low degree of recombination, while strains CBS 162.54, CBS 406.96, CBS 161.54 (Australia), CBS 100434 (Madagascar) and CBS 131844, CBS 131839 (Japan) and CBS 362.70, 858.96 (Venezuela) were connected by a network suggestive for recombination.

The split decomposition network showed that strains CBS 162.54, 406.96, 161.54 (Australia), 100434 (Madagascar) and 131844, 131839 (Japan) and 362.70, 858.96 (Venezuela) were connected by a network structure, suggesting recombination could be occurring among strains.

Performing these tests on the *Fonsecaea* dataset (S3 Table), recombination signals were detected only in ITS by max χ^2^ (*P* = 0.00). Gard and PHI did not find statistically significant evidence of recombination in both datasets of *BT2* (PHI: *P* = 0.21; Gard negative) and ITS (PHI: *P* = 0.82; Gard negative). This was confirmed by the split decomposition network (S5 Fig), where three separated groups were revealed. The split decomposition network of *Fonsecaea* showed a different history than the one found in *C*. *carrionii*, although the *F*. *nubica*-group showed a network with CBS 269.64 (Africa) and CBS 444.62 (Surinam), and the *F*. *monophora*-group with SUMS 0322, SUMS 0324, CBS 121722 and 121723 (all from Asia).

## Discussion

*Cladophialophora carrionii* is one of the major agents of human chromoblastomycosis, a chronic and severely mutilating skin disorder. The disease has only three consistent phylogenetic groups of etiological agents (S1 Fig). One of these is *Rhinocladiella aquaspersa*, taking an isolated position. Three *Fonsecaea* agents (*F*. *monophora*, *F*. *nubica*, and *F*. *pedrosoi*) compose a monophyletic clade amidst *Cladophialophora* species causing none or other disorders. The two *Cladophialophora* agents, *C*. *carrionii* and *C*. *samoensis* cluster separately in a well-supported ‘carrionii-clade’. In the carrionii-clade *C*. *carrionii* is prevalent, and the rare species *C*. *samoensis* is only known from a single strain on the Pacific island Samoa [11]. The clade also comprises some species with other etiologies, as well as *Phialophora verrucosa* and *P*. *americana*, both only occasionally having been reported from cases of chromoblastomycosis. All agents of the disease belong to the order *Chaetothyriales* comprising the pathogenic black yeasts and their filamentous relatives. Thus, within the *Chaetothyriales* independent evolution of agents of chromoblastomycosis has taken place, the agents being polyphyletic but sharing basic traits of the order (S1 Fig). In general, members of *Chaetothyriales* inhabit environments that are toxic or poor in nutrients, while many are opportunistic agents of diseases other than chromoblastomycosis.

Most agents of chromoblastomycosis are found in endemic areas in humid tropical climate zones, but *C*. *carrionii* is exceptional in being restricted to arid desert-like climates inhabited by cactus plants. *Cladophialophora carrionii* had greater number of haplotypes than individual species of *Fonsecaea* (Table 1). The high haplotype diversity and low nucleotide diversity values for *C*. *carrionii* populations indicated small differences between haplotypes. This matched with the number of polymorphic sites (*S*) which mostly represented singleton mutations. The combination of high haplotype and low nucleotide diversity suggests a rapid population expansion or a small effective population size as observed in earlier studies [25]. The overall negative values of Tajima’s *D* test and Fu & Li’s D* and *F*_ST_* tests indicate an excess of rare mutations in populations, which may imply a recent population expansion of *C*. *carrionii* populations.

The haplotype network of *Cladophialophora carrionii* using ITS and *BT2* (Fig 1) shows three approximate groups (cA, cB, cC; Fig 1), which show high degrees of geographic structuring, either from South America, Asia, or Australia, respectively, with African strains being dispersed. The genomic fingerprints of *C*. *carrionii* generated by AFLP (S3 Fig) show a similar picture: South American (AFLP-A), Asian (AFLP-B) and Australian (AFLP-C) clusters are recognized, with some scattered African strains. No clear separation of groups cA and cB is apparent at the species level (Fig 1); only the poorly populated group cC is distinct. The clusters of Australia, Asia and South America are relatively homogeneous with high membership coefficients, in contrast to the cluster of African haplotypes which has a high level of admixture (Fig 3). Furthermore, population differentiations were evident among subpopulations in Africa, Asia and Central/South America. The African population has relationships to Asian, South American, and Australia populations as suggested by low and non-significant *F*_*ST*_ values (Table 3). In general this suggests a slow vector of dispersal, with oceans functioning as barriers and could be attributed to founder effects or selection [26]. High levels genetic differentiation correlates with high levels of endemism. Gene flow across continents is limited as the fungus is dispersed through conidia that are reluctantly airborne and cannot travel over long distances [10]. Split decomposition analysis of *C*. *carrionii* loci revealed evidence of recombination (Fig 4, S3 Table), suggesting that cryptic sexual reproduction may be present.

The presence of multiple, divergent haplotypes in the African population may indicate that Africa is the source of the pathogen. One of the lineages may have migrated and have given rise to Asian and South American populations recently. Although the fungus should be able to reproduce sexually, clonal expansion of a major haplotype has taken place, as is particularly obvious in South America. *Cryptococcus neoformans* var. *grubii* in South Africa also has geographically restricted, genetically diverse population [27].

The evolutionary history and genetic structure of the *Fonsecaea* clade shows a different picture with a tri-partition of clearly individualized species [24] each comprising only a few haplotypes (Fig 2). The differences have remained distinct in multilocus analyses [28]. AFLP profiles were concomitant with these data [24]. The three recognized species have separated long time ago and they are reproductively isolated given the variation accumulated among the haplotypes (Fig 2). *Fonsecaea pedrosoi* is highly clonal and consists of a single AFLP group [24]. The species is nearly endemic to South America. *Fonsecaea nubica* has a global distribution and more divergent haplotypes, possibly explained by accumulation of deleterious mutations with preponderant clonal reproduction. *Fonsecaea monophora* shows clonal expansion in Asia and South America and may have gone through recent divergence, with allopatric speciation between Asian and American populations. Overall, a smaller amount of haplotypes is found in a comparable set of strains of three *Fonsecaea* species than in the single species *C*. *carrionii*, but average diversity (*N*_*d*_) of the *Fonsecaea* clade is 2.5 times larger than that of *C*. *carrionii*. Diversity of *C*. *carrionii* is largely due to singletons, whereas the *Fonsecaea* spp. dataset contained mainly fixed mutations [28] (Table 1). Fixation of the three *Fonsecaea* spp. is likely to be significantly older than the divergence noted in *C*. *carrionii*.

The structured distribution of agents of chromoblastomycosis is remarkable. *Cladophialophora samoënsis* is found thus far on a single Polynesian island only [11], *Rhinocladiella aquaspersa* is preponderantly found in South America [15,16], and the species discussed in this paper show significant genetic differences between continents. Most other opportunists known in the order *Chaetothyriales* investigated thus far lack geographic structuring. Sudhadham et al. [29] analyzed worldwide populations of the black yeast *Exophiala dermatitidis*. The species has several sequence types, which all showed random distribution, and no structuring was observed in AFLP data. Possibly this species was originally distributed by frugivorous animals along the equator before it entered the human indoor environment [30]. Our data on agents of chromoblastomycosis suggest that another type distribution is concerned. Despite the apparent differences in evolution, both *C*. *carrionii* and *Fonsecaea* spp. populations are structured, African populations showing largest diversities. On the other hand, *C*. *carrionii* and *Fonsecaea* spp. differ by lineage sorting, which has led to three reproductively isolated species in *Fonsecaea*. The possibility that animal hosts play a role in the evolution of agents of chromoblastomycosis is not excluded, but environmental infection probably is more significant: two strains from cactus debris in Venezuela, CBS 861.96, CBS 862.96 and CBS 863.96 (their presence indicated with an asterisk in Figs 1 and S3) were identical to clinical strains from the same area.

We conclude that these agents of human disease have diverse distribution patterns and population dynamics even though they share a common ancestor within the order *Chaetothyriales*. It may be speculated that they have taken advantage of and adapted to human hosts as a new ecological niche.

## Materials and Methods

### Fungal Strains and Culture Conditions

Strains were analyzed in this study including 81 strains of *Chaetothyriales* (family *Herpotrichiellacae*), 73 strains of *Cladophialophora carrionii* and 60 strains of *Fonsecaea* species. Cultures originated from the Centraalbureau voor Schimmelcultures (CBS-KNAW Fungal Biodiversity Centre, Utrecht, The Netherlands). Additional strains were acquired from the collection in Institut Pasteur (Paris, France). Strains were listed in Supplementary material (S1 and S2 Tables). Stock cultures were maintained on slants of 4% malt extract agar and oatmeal agar at 24°C.

### DNA Extraction and Sequencing

DNA extraction was performed as described previously [31]. The quality of genomic DNA was verified on agarose gels. Two loci, ITS region of rDNA and the partial ß-tubulin (*BT2*) gene, were amplified and sequenced for all strains using primer pairs ITS1/4 and *Bt2a/Bt2b*, respectively [32].

### Data Analysis

ITS Sequences were collected using BioNumerics v. 4.5 (Applied Maths, Sint-Martens-Latem, Belgium) and alignments were made using Muscle v. 3.8 [33]. ITS trees were reconstructed using MEGA5 [34] with the neighbour joining algorithm with Kimura 2 correction with 100 bootstrap replications. DnaSP v. 5.10 [35] was used to determine the extent of DNA polymorphism, such as number of polymorphic sites (*S*), haplotype diversity (*H*_d)_, and nucleotide diversity (π). We also tested neutrality by Tajima’s *D* test as well as Fu & Li’s D* and F* test using DnaSP v. 5.10. Negative values of these neutrality tests suggest evidence for the excess of high-frequency variants relative to expectation, indicating population size expansion (e.g., after a bottleneck or a selective sweep) and/or purifying selection, whereas positive values suggest evidence for balancing or dominant selection or expansion of rare polymorphisms. Genetic differentiation among populations (*F*_st)_ based on the haplotypes created for different geographical regions in each fungal species was estimated using Slatkin linearized *F*_*st*_ statistics. Strains from Europe and the U.S.A. were discarded from the *F*_*st*_ analysis because of small sample sizes.

Structure v. 2.3 [36] was used as a model-based clustering method to analyze the association of individual strains and to infer potential genetic clusters with estimation on the level of admixture using the haplotypes generated from DnaSP. Structure estimates allele frequencies in each group and population relationships for every individual given the number of clusters (*K*) [37]. It also uses a Monte Carlo Markov Chain (MCMC) to group individuals into *K* distinct populations that minimize Hardy-Weinberg disequilibrium between loci within groups by including prior information, for example, the geographical location of populations [37]. The number of clusters *(K*) was defined from 1 to 10, and each run was conducted with the admixture and correlated allele frequency model, with 100,000 MCMC generations burn-in period followed by 900,000 MCMC generations. Ten independent runs were performed, and the delta log likelihood value for each *K* was determined to ensure consistency among runs. The spatial sampling location of each population was included as prior. We used Structure Harvester (http://taylor0.biology.ucla.edu/structureHarvester) to estimate the optimal value of *K* using the *ΔK* method (rate changes in the log likelihood) described in [38].

The level of recombination was investigated using various approaches. First, two analytical tools utilizing maximum chi-square test (max χ^2^: www.lifesci.sussex.ac.uk/CSE/test/maxchi.php) and Gard (Genetic Analysis for Recombination Detection [39]) were performed on ITS and *BT2* data sets of *C*. *carrionii* and *Fonsecaea* species, respectively. Within each data set, a sliding window was generated on each possible pair of sequences. Chi-squared statistics were computed to compare the proportion of identical sites within the left half-window with the proportion of identical sites within the right half-window. Recombination is likely when there is a significant discrepancy between the two proportions. The maximal chi-squared value over all sequence pairs was inferred as evidence of recombination.

SplitsTree v. 4.8 [35] was also used to perform the Pairwise Homoplasy Index (PHI) test on ITS and *BT2* data separately and to build split decomposition trees on concatenated (ITS-*BT2*) data of *C*. *carrionii* and *Fonsecaea* spp. data sets, respectively; a recombination event would create a homoplasious region in the sequence. PHI compares topology-informative sites in a pairwise matrix measuring the compatibility between closely linked sites using a refined incompatibility matrix and a refined incompatibility score. Max χ^2^ is permutating the columns of the alignment while PHI is using parsimoniously informative sites. We used these methods simultaneously as they have different prior distributions and assumptions and we wished to compare and evaluate the robustness of data for detecting recombination events among populations.

### AFLP Fingerprinting

Amplified Fragment Length Polymorphism (AFLP) analyses were performed by using 100–200 ng DNA. Five μl DNA was added to 15 μl restriction and ligation mixture containing 1 U of T4 DNA ligase buffer (Promega, Leiden, The Netherlands), 50 pmol HpyCH4IV adapter, 50 pmol MseI adapter, 2 U HpyCH4IV (New England Biolabs, Beverly, MA, U.S.A.), and 2 U MseI (New England Biolabs). The restriction ligation mixture was incubated for 1 h at 20°C and diluted five times with 10 mM Tris-HCl (pH 8.3) buffer. Adapters were made by mixing equimolar amounts of complementary oligonucleotides (5’-CTCGTAGACTGCGTACC-3’ and 5’-CGGGTACGCAGTC-3’ for HpyCH4IV; 5’-GACGATGAGTCCTGAC-3’ and 5’-TAGTCAGGACTCAT-3’ for MseI) and heated to 95°C for 1 min, subsequently cooled slowly to ambient temperature. Prior further handing, the restriction-ligation reaction was terminated by the addition of 80 μl 10 mM Tris-HCl. One microliter of this diluted restriction-ligation mixture was used for amplification in a volume of 25 μl under the following conditions: 1 μM HpyCH4IV primer with one selective residue (underlined) (5’-Flu-GTAGACTGCGTACCCGT**C**-3’), 1 μM MseI primer with four selective residues (underlined) (5’-GATGAGTCCTGACTAA**TGAG**-3’), 0.2 mM of each deoxynucleoside triphosphate, and 1 U of Taq DNA polymerase (Roche Diagnostics, Almere, The Netherlands) in 1× reaction buffer containing 1.5 mM MgCl_2_. Amplification was done as follows. After an initial denaturation step for 4 min at 94°C in the first 20 cycles, a touchdown procedure was applied: 15 s of denaturation at 94°C, 15 s of annealing at 66°C, with the temperature for each successive cycle lowered by 0.5°C, and 1 min of extension at 72°C. Cycling was then continued for a further 30 cycles with an annealing temperature of 56°C. After completion of the cycles, incubation at 72°C for 10 min was performed before the reaction mixtures were cooled to room temperature. The amplicons were then 10× diluted, and 1 μl was added to a mixture of 8.75 μl water and 0.25 μl ET400-R size standard (GE Healthcare, Diegem, Belgium) and analyzed on a Mega BACE 500 automated DNA platform (GE Healthcare) according to the manufacturer’s instructions.

## Supporting Information

S1 Fig Neighbor joining tree of ITS sequences of genera belonging to the order *Chaetothyriales*. The substitution model used is Kimura 2-parameter model with Gamma correction and 1000 bootstrap replicates. Values show bootstrap support >70%. The red branches contain agents causing chromoblastomycosis. Dotted line marks the groups of fungi where occasional strains from chromoblastomycosis have been reported.
(TIF)

S2 Fig Haplotype groups alignment in *Cladophialophora carrionii* and *Fonsecaea* spp. The concatenated dataset (ITS-*BT2*) had a total of 35 haplotypes in *C*. *carrionii* and 18 haplotypes in *Fonsecaea* spp.
(TIF)

S3 Fig Profiles generated by AFLP of *Cladophialophora carrionii*. AFLP analysis of 73 strains of *C*. *carrionii* revealed three main groups marked as AFLP-A, AFLP-B, AFLP-C, AFLP-D.
(TIF)

S4 Fig The K-value calculated by Structure 2.3.4 for *Cladophialophora carrionii* and *Fonsecaea* spp. The *C*. *carrionii* data set is subdivided in 4 groups according to the graph. The *Fonsecaea* spp. data set is subdivided in 3 groups according to the graph.
(TIF)

S5 Fig Split decomposition network of *Fonsecaea* spp. constructed with Splitstree. The split decomposition network of *Fonsecaea* spp. showed three separated groups, although the *F*. *nubica*—group showed a network with CBS 269.64 (Africa) and CBS 444.62 (Surinam), and the *F*. *monophora*—group with SUMS 0322, SUMS 0324, CBS 121722 and 121723 (all from Asia).
(TIF)

S1 Table Strains of *Cladophialaphora carrionii* used in this study. (PDF)

S2 Table Strains of *Fonsecaea* spp. used in this study. (PDF)

S3 Table Summary of Max χ^2^, GARD and PHI based on the ITS and *BT2* data in *Cladophialophora carrionii* and *Fonsecaea* spp. (PDF)

S1 Text The list of strains and cited number presented in the Fig 4. (DOC)
