# Supplementary figures and images for: Global Spread of Human Chromoblastomycosis Is Driven by Recombinant Cladophialophora carrionii and Predominantly Clonal Fonsecaea Species
Source: PLoS Negl Trop Dis. 2015 Oct 23;9(10):e0004004. doi: 10.1371/journal.pntd.0004004 (PMC4619687; doi:10.1371/journal.pntd.0004004)

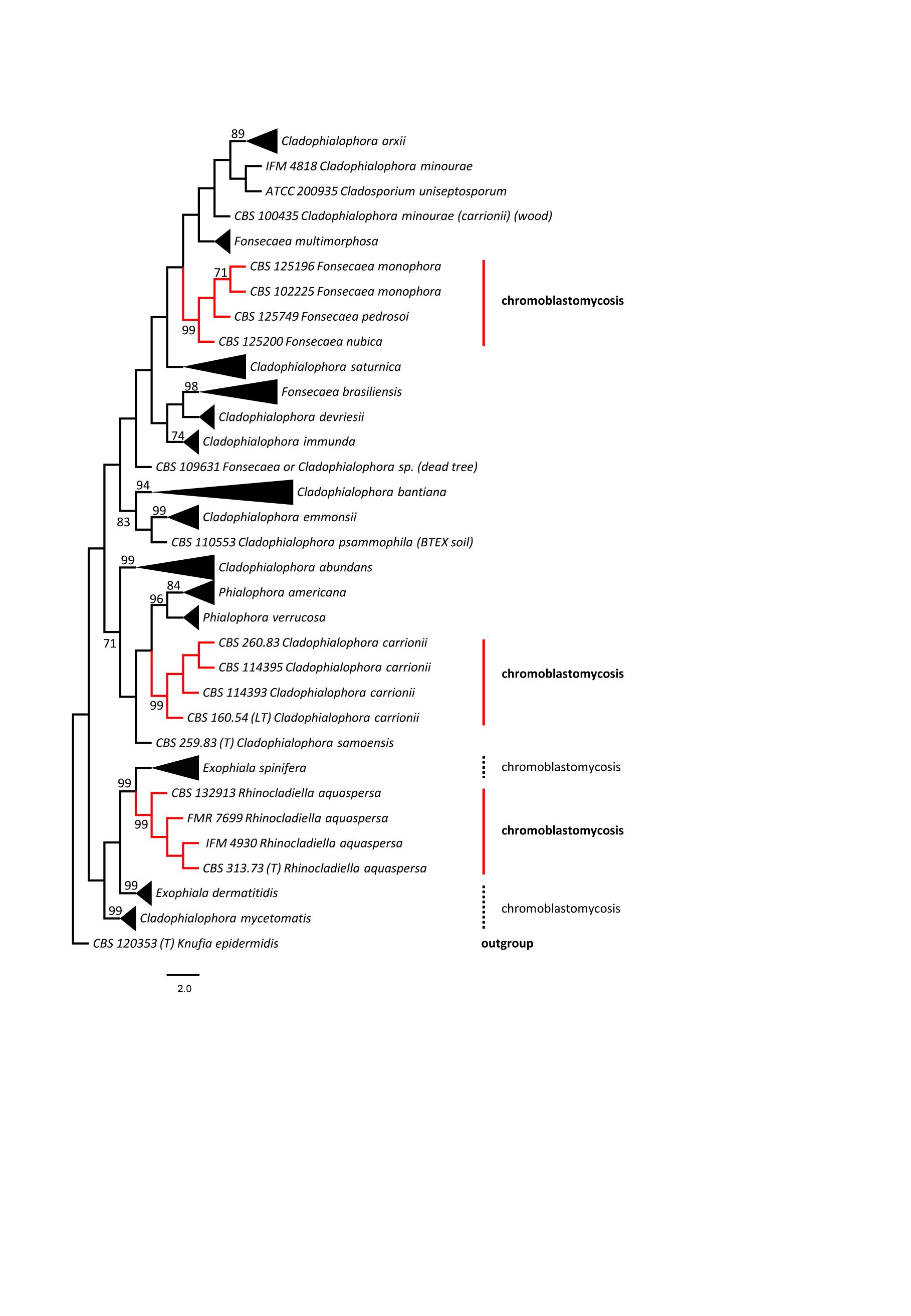

Supplement: S1 Fig — The substitution model used is Kimura 2-parameter model with Gamma correction and 1000 bootstrap replicates. Values show bootstrap support >70%. The red branches contain agents causing chromoblastomycosis. Dotted line marks the groups of fungi where occasional strains from chromoblastomycosis have been reported. (TIF) [file pntd.0004004.s001.tif]

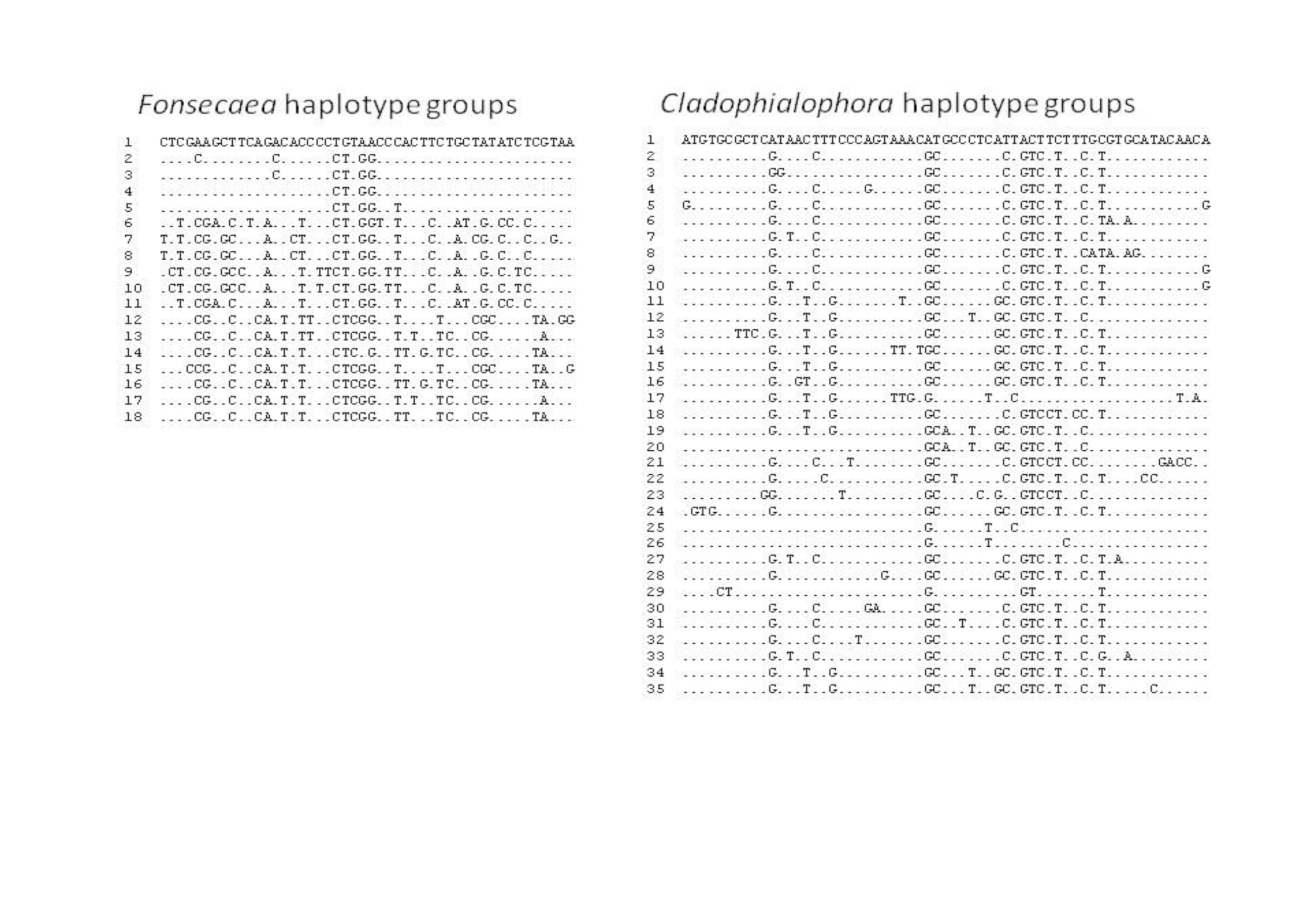

Supplement: S2 Fig — The concatenated dataset (ITS-BT2) had a total of 35 haplotypes in C. carrionii and 18 haplotypes in Fonsecaea spp. (TIF) [file pntd.0004004.s002.tif]

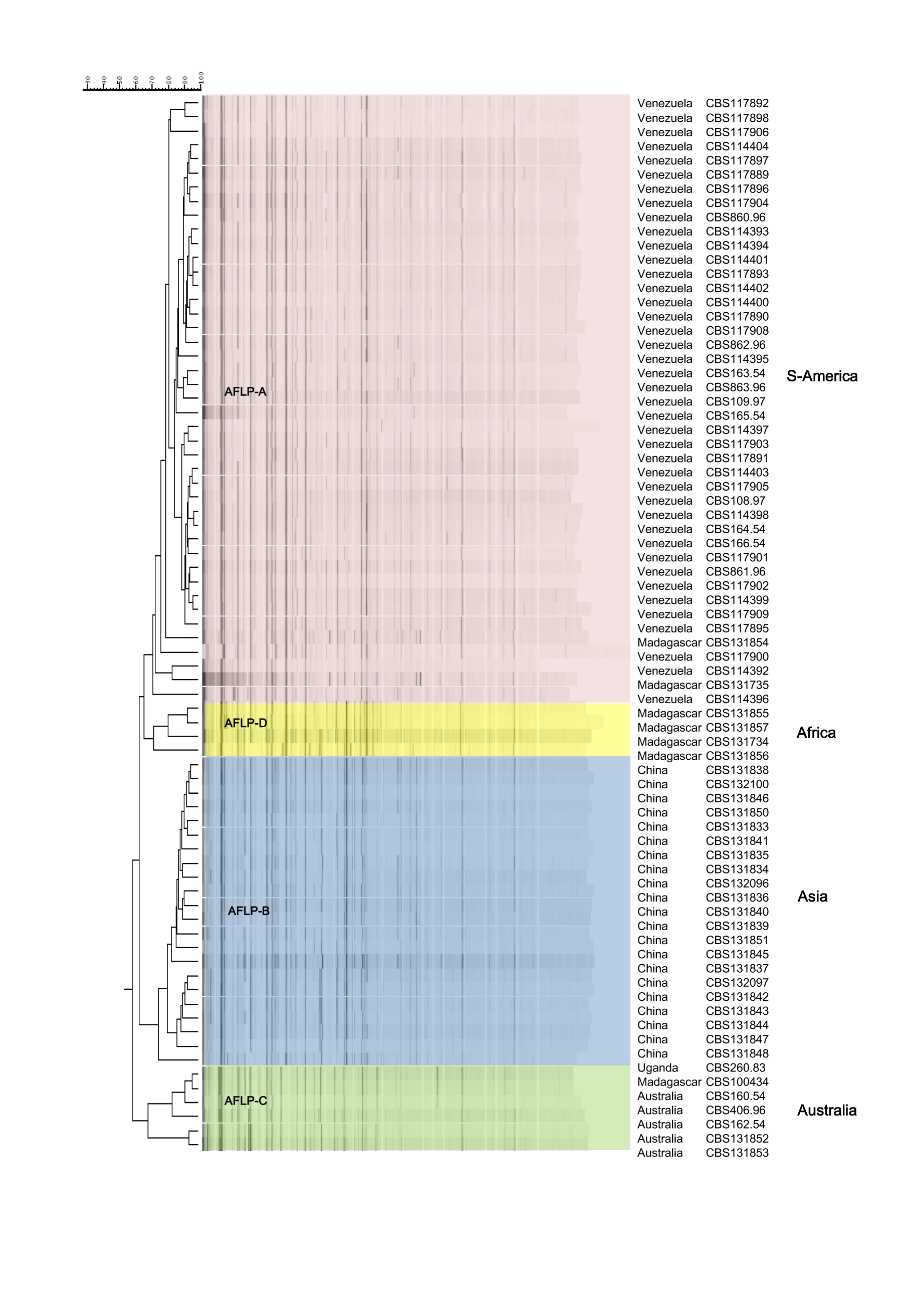

Supplement: S3 Fig — AFLP analysis of 73 strains of C. carrionii revealed three main groups marked as AFLP-A, AFLP-B, AFLP-C, AFLP-D. (TIF) [file pntd.0004004.s003.tif]

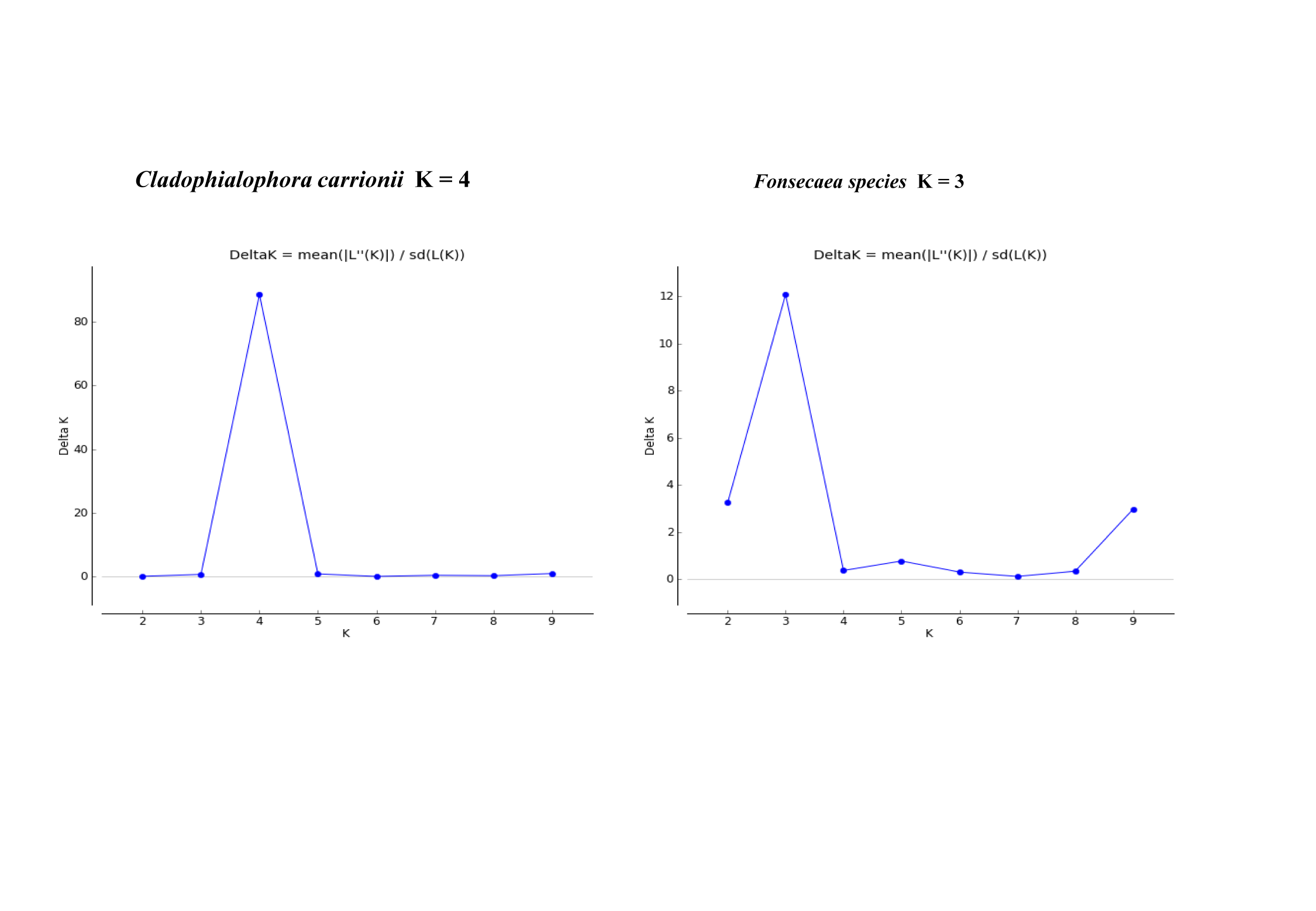

Supplement: S4 Fig — The C. carrionii data set is subdivided in 4 groups according to the graph. The Fonsecaea spp. data set is subdivided in 3 groups according to the graph. (TIF) [file pntd.0004004.s004.tif]

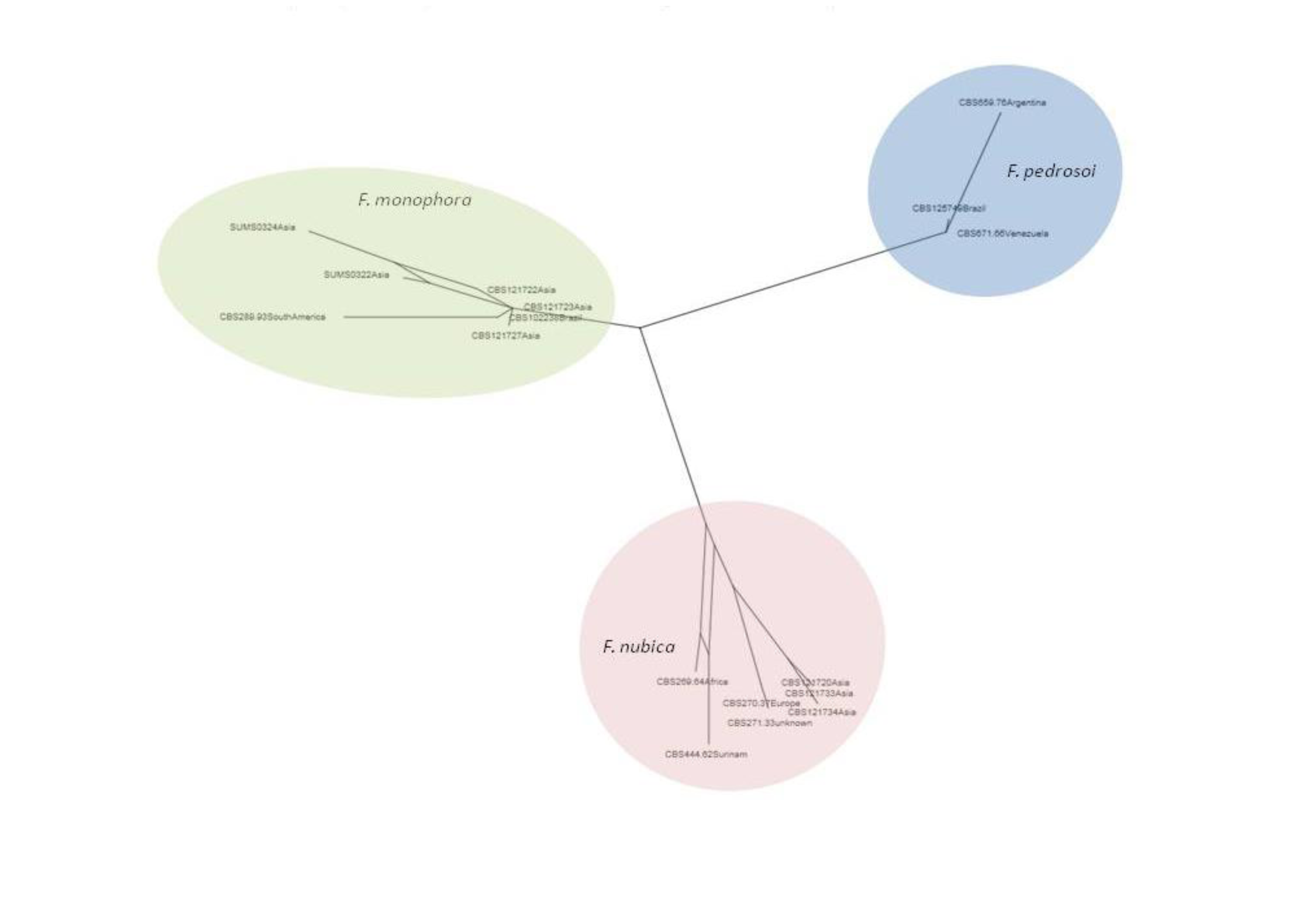

Supplement: S5 Fig — The split decomposition network of Fonsecaea spp. showed three separated groups, although the F. nubica—group showed a network with CBS 269.64 (Africa) and CBS 444.62 (Surinam), and the F. monophora—group with SUMS 0322, SUMS 0324, CBS 121722 and 121723 (all from Asia). (TIF) [file pntd.0004004.s005.tif]
