# Supplementary material for: Global Spread of Human Chromoblastomycosis Is Driven by Recombinant Cladophialophora carrionii and Predominantly Clonal Fonsecaea Species
Source: PLoS Negl Trop Dis. 2015 Oct 23;9(10):e0004004. doi: 10.1371/journal.pntd.0004004 (PMC4619687; doi:10.1371/journal.pntd.0004004)
Supplement: S1 Table — (PDF) [file pntd.0004004.s006.pdf]

S1Table Strains used in this study

Name	Strains no.	Source	Origin	Accession no.-BT2	Accession no.-ITS
Cladophialophora carrionii	CBS 108.97	Chromoblastomycosis, male	Venezuela	EU137188.1	EU137306.1
Cladophialophora carrionii	CBS 109.97	Chromoblastomycosis, male	Venezuela	-	-
Cladophialophora carrionii	CBS 164.54	Chromoblastomycosis, male	Venezuela	-	-
Cladophialophora carrionii	CBS 165.54	Chromoblastomycosis, male	Venezuela	EU137187.1	EU137305.1
Cladophialophora carrionii	CBS 166.54	Chromoblastomycosis, male	Venezuela	EU137173.1	EU137290.1
Cladophialophora carrionii	CBS 114392	Chromoblastomycosis, female	Venezuela	EU137150.1	EU137267.1
Cladophialophora carrionii	CBS 114393	Chromoblastomycosis, male	Venezuela	EU137151.1	EU137268.1 KF928452.1
Cladophialophora carrionii	CBS 114394	Chromoblastomycosis, male	Venezuela	-	EU137307.1
Cladophialophora carrionii	CBS 114395	Chromoblastomycosis, female	Venezuela	EU137182.1	EU137299.1
Cladophialophora carrionii	CBS 114396	Chromoblastomycosis, male	Venezuela	EU137152.1	EU137269.1
Cladophialophora carrionii	CBS 114397	Chromoblastomycosis, male	Venezuela	EU137153.1	EU137270.1
Cladophialophora carrionii	CBS 114398	Chromoblastomycosis, female	Venezuela	EU137154.1	EU137271.1
Cladophialophora carrionii	CBS 114399	Chromoblastomycosis, female	Venezuela	EU137155.1	EU137272.1
Cladophialophora carrionii	CBS 114400	Chromoblastomycosis, male	Venezuela	EU137156.1	EU137273.1
Cladophialophora carrionii	CBS 114401	Chromoblastomycosis, female	Venezuela	EU137157.1	EU137274.1
Cladophialophora carrionii	CBS 114402	Chromoblastomycosis, female	Venezuela	EU137158.1	EU137275.1
Cladophialophora carrionii	CBS 114403	Chromoblastomycosis, male	Venezuela	EU137159.1	EU137276.1
Cladophialophora carrionii	CBS 114404	Chromoblastomycosis, male	Venezuela	EU137196.1	EU137311.1
Cladophialophora carrionii	CBS 117889	Chromoblastomycosis, female	Venezuela	-	EU137280.1
Cladophialophora carrionii	CBS 117890	Chromoblastomycosis, male	Venezuela	-	EU137279.1
Cladophialophora carrionii	CBS 117891	Chromoblastomycosis, male	Venezuela	-	EU137278.1
Cladophialophora carrionii	CBS 117893	Chromoblastomycosis, male	Venezuela	EU137200.1	EU137316.1
Cladophialophora carrionii	CBS 117895	Chromoblastomycosis, male	Venezuela	EU137169.1	EU137286.1
Cladophialophora carrionii	CBS 117896	Chromoblastomycosis, male	Venezuela	-	EU137285.1
Cladophialophora carrionii	CBS 117897	Chromoblastomycosis, male	Venezuela	-	EU137314.1

Cladophialophora carrionii	CBS 117900	Chromoblastomycosis, male	Venezuela	-	EU137284.1
Cladophialophora carrionii	CBS 117901	Chromoblastomycosis, female	Venezuela	EU137197.1	EU137312.1
Cladophialophora carrionii	CBS 117902	Chromoblastomycosis, male	Venezuela	-	EU137283.1
Cladophialophora carrionii	CBS 117903	Chromoblastomycosis, male	Venezuela	-	EU137282.1
Cladophialophora carrionii	CBS 117904	Chromoblastomycosis, male	Venezuela	-	EU137281.1
Cladophialophora carrionii	CBS 117905	Chromoblastomycosis, male	Venezuela	-	EU137300.1
Cladophialophora carrionii	CBS 117906	Chromoblastomycosis, male	Venezuela	EU137171.1	EU137288.1
Cladophialophora carrionii	CBS 117908	Chromoblastomycosis, male	Venezuela	EU137191.1	-
Cladophialophora carrionii	CBS 117909	Chromoblastomycosis, male	Venezuela	EU137170.1	EU137287.1
Cladophialophora carrionii	CBS 860.96	Dry plant debris	Venezuela	-	-
Cladophialophora carrionii	CBS 861.96	Dry plant debris	Venezuela	EU137194.1	EU137309.1
Cladophialophora carrionii	CBS 862.96	Dry plant debris	Venezuela	EU137199.1	EU137315.1
Cladophialophora carrionii	CBS 863.96	Dry plant debris	Venezuela	EU137179.1	EU137296.1 KP131829.1
Cladophialophora carrionii	CBS131833	Chromoblastomycosis, male	China	-	-
Cladophialophora carrionii	CBS131834	Chromoblastomycosis, male	China	-	-
Cladophialophora carrionii	CBS131835	Chromoblastomycosis, male	China	-	-
Cladophialophora carrionii	CBS131836	Chromoblastomycosis, male	China	-	-
Cladophialophora carrionii	CBS131838	Chromoblastomycosis, male	China	-	-
Cladophialophora carrionii	CBS131839	Chromoblastomycosis, male	China	-	-
Cladophialophora carrionii	CBS131840	Chromoblastomycosis, male	China	-	-
Cladophialophora carrionii	CBS131841	Chromoblastomycosis, male	China	-	-
Cladophialophora carrionii	CBS131842	Chromoblastomycosis, male	China	-	-
Cladophialophora carrionii	CBS131843	Chromoblastomycosis, male	China	-	-
Cladophialophora carrionii	CBS131844	Chromoblastomycosis, male	China	-	-
Cladophialophora carrionii	CBS131845	Chromoblastomycosis, male	China	-	-
Cladophialophora carrionii	CBS131846	Chromoblastomycosis, male	China	-	-

Cladophialophora carrionii	CBS131847	Chromoblastomycosis, male	China	-	-
Cladophialophora carrionii	CBS131848	Chromoblastomycosis, male	China	-	-
Cladophialophora carrionii	CBS131850	Chromoblastomycosis, male	China	-	-
Cladophialophora carrionii	CBS131851	Chromoblastomycosis, male	China	-	-
Cladophialophora carrionii	CBS132096	Chromoblastomycosis, male	China	-	-
Cladophialophora carrionii	CBS132097	Chromoblastomycosis, male	China	-	-
Cladophialophora carrionii	CBS132100	Chromoblastomycosis, male	China	-	-
Cladophialophora carrionii	CBS131837	Chromoblastomycosis, male	China	-	-
Cladophialophora carrionii	CBS131854	Chromoblastomycosis -	Madagascar	-	-
Cladophialophora carrionii	CBS131855	Chromoblastomycosis -	Madagascar	-	-
Cladophialophora carrionii	CBS131856	Chromoblastomycosis -	Madagascar	-	-
Cladophialophora carrionii	CBS131734	Chromoblastomycosis -	Madagascar	-	-
Cladophialophora carrionii	CBS131735	Chromoblastomycosis -	Madagascar	-	-
Cladophialophora carrionii	CBS131857	Chromoblastomycosis -	Madagascar	-	-
Cladophialophora carrionii	CBS 100434	Chromoblastomycosis, male	Madagascar	EU137172.1	EU137289.1
Cladophialophora carrionii	CBS 260.83	Chromoblastomycosis, male	Uganda	EU137175.1	EU137292.1
Cladophialophora carrionii	CBS 160.54	Chromoblastomycosis, male	Australia	EU137201.1	EU137266.1 KF928453.1
Cladophialophora carrionii	CBS 162.54	Chromoblastomycosis, male	Australia	EU137185.1	EU137303.1 KF928451.1
Cladophialophora carrionii	CBS 163.54	Chromoblastomycosis, male	venezuela	EU137186.1	EU137304.1
Cladophialophora carrionii	CBS131852	Unknown -	Australia	-	-
Cladophialophora carrionii	CBS131853	Unknown -	Australia	-	-
Cladophialophora carrionii	CBS 406.96	Chromoblastomycosis, male	Australia	EU137202.1	EU137317.1
