# Supplementary material for: Global Spread of Human Chromoblastomycosis Is Driven by Recombinant Cladophialophora carrionii and Predominantly Clonal Fonsecaea Species
Source: PLoS Negl Trop Dis. 2015 Oct 23;9(10):e0004004. doi: 10.1371/journal.pntd.0004004 (PMC4619687; doi:10.1371/journal.pntd.0004004)
Supplement: S2 Table — (PDF) [file pntd.0004004.s007.pdf]

S2Table Strains used in this study

Name	Strains no.	Source	Origin	Accesion no.-BT2	Accesion no.-ITS
nsecaea pedrosoi	CBS 342.34	Chromoblastomycosis, man	Brazil	-	EU938589.1
Fonsecaea pedrosoi	CBS 122741	Chromoblastomycosis, man foot	Mexico	-	EU938590.1
Fonsecaea pedrosoi	CBS 122740	Chromoblastomycosis, man foot	Mexico	-	-
Fonsecaea pedrosoi	CBS 273.66	Mouse passage	Venezuela	EU938565.1	EU938587.1
Fonsecaea pedrosoi	CBS 274.66	Mouse passage	Venezuela	-	-
Fonsecaea pedrosoi	CBS 253.49	Chromoblastomycosis, man	Uruguay	EU938571.1	AY366919.1
Fonsecaea pedrosoi	CBS 102247	Chromoblastomycosis, man	Brazil	-	-
Fonsecaea pedrosoi	CBS 102245	Chromoblastomycosis, man	Brazil	EU938562.1	-
Fonsecaea pedrosoi	CBS 201.31	Auditory canal, gazelle	Libiya	EU938563.1	-
Fonsecaea pedrosoi	CBS 670.66	Mouse passage, soil	Venezuela	EU938564.1	-
Fonsecaea pedrosoi	CBS 122736	Chromoblastomycosis, man	Mexico	-	-
Fonsecaea pedrosoi	CBS 285.47	Chromoblastomycosis, man	Puerto Rico	EU938573.1	-
Fonsecaea pedrosoi	CBS 102244	Chromoblastomycosis, man	Brazil	-	-
Fonsecaea pedrosoi	CBS 271.37	Chromoblastomycosis, man	South America	KN846971.1	AY366920.1
Fonsecaea pedrosoi	CBS 659.76	Chromoblastomycosis, man	Argentina	-	KC886423.1
Fonsecaea pedrosoi	CBS 125749	Chromoblastomycosis, man	Brazil	-	-
Fonsecaea pedrosoi	CBS 212.77	Chromoblastomycosis, man	Netherlands	EU938568.1	AY366917.1
Fonsecaea pedrosoi	CBS 272.37	Chromoblastomycosis, man	Brazil	-	-
Fonsecaea pedrosoi	CBS 122849	Chromoblastomycosis, man	Mexico	-	-
Fonsecaea pedrosoi	CBS 117910	Chromoblastomycosis, human	Venezuela	-	EU938595.1
Fonsecaea pedrosoi	CBS 102224	Wood, Grevillea, plant	Brazil	-	FJ785471.1
Fonsecaea monophora	CBS 123849	Chromoblastomycosis, man	Africa	-	-
Fonsecaea monophora	CBS 397.48	Chromoblastomycosis, man	Brazil	EU938555.1	EU938579.1
Fonsecaea monophora	CBS 102243	Chromoblastomycosis, man	Brazil	-	-
Fonsecaea monophora	CBS 102246	Chromoblastomycosis, man	Brazil	EU938543.1	-

Fonsecaea monophora	CBS 102248	Chromoblastomycosis, man	Brazil	EU938550.1	AY857511.1
Fonsecaea monophora	CBS 269.37	Chromoblastomycosis, man	South America	-	EU938584.1
Fonsecaea monophora	CBS 117542	Brain, biopsy, man	USA	-	-
Fonsecaea monophora	CBS 117237	Chromoblastomycosis, man leg	USA	EU938544.1	-
Fonsecaea monophora	CBS 115830	Brain, human	Brazil	EU938548.1	-
Fonsecaea monophora	CBS 121725	Chromoblastomycosis, human	China	-	-
Fonsecaea monophora	CBS 289.93	lymphnode, seabear	Netherlands	EU938554.1	-
Fonsecaea monophora	CBS 121721	Chromoblastomycosis, human	China	-	-
Fonsecaea monophora	CBS 121722	Chromoblastomycosis, human	China	-	-
Fonsecaea monophora	CBS 121723	Chromoblastomycosis, human	China	-	-
Fonsecaea monophora	CBS 121724	Chromoblastomycosis, human	China	-	-
Fonsecaea monophora	CBS 121726	Chromoblastomycosis, human	China	-	-
Fonsecaea monophora	CBS 121727	Chromoblastomycosis, human	China	-	-
Fonsecaea monophora	CBS 121728	Chromoblastomycosis, human	China	-	-
Fonsecaea monophora	CBS 121729	Chromoblastomycosis, human	China	-	-
Fonsecaea monophora	CBS 121730	Chromoblastomycosis, human	China	-	--
Fonsecaea monophora	CBS 121731	Chromoblastomycosis, human	China	-	-
Fonsecaea monophora	CBS 121732	Chromoblastomycosis, human	China	-	-
Fonsecaea monophora	CBS 117238	Brain, human	UK	-	-
Fonsecaea monophora	CBS 102238	Soil	Brazil	EU938546.1	-
Fonsecaea monophora	CBS 102242	Chromoblastomycosis, man	Brazil	EU938549.1	-
Fonsecaea monophora	CBS 117236	Brain abscess, man	USA	EU938551.1	-
Fonsecaea monophora	SUMS 0300	Chromoblastomycosis, human	China	EU938556.1	-
Fonsecaea monophora	SUMS 0324	Chromoblastomycosis, human	China	EU938557.1	-
Fonsecaea monophora	SUMS 0322	Chromoblastomycosis, human	China	EU938558.1	EU938581.1
Fonsecaea monophora	CBS 102229	Decaying vegetable cover, plant	Brazil	-	-

Fonsecaea nubica	CBS 121733	Chromoblastomycosis, human	China	-	-
Fonsecaea nubica	CBS 444.62	Chromoblastomycosis, human	Surinam	EU938575.1	KP132198.1
Fonsecaea nubica	CBS 121720	Chromoblastomycosis, human	China	-	NR_111333.1
Fonsecaea nubica	CBS 269.64	Chromoblastomycosis, human	Africa	-	-
Fonsecaea nubica	CBS 121734	Chromoblastomycosis, human	China	-	-
Fonsecaea nubica	CBS 271.33	Chromoblastomycosis, human	South America	EU938578.1	-
Fonsecaea nubica	CBS 270.37	Unknown	France	-	EU938594.1
Fonsecaea nubica	CBS 277.29	Chromoblastomycosis, man	Brazil	-	EU938593.1
Fonsecaea nubica	CBS 557.76	Unknown	Unknown	-	-
