# Supplementary material for: Global Spread of Human Chromoblastomycosis Is Driven by Recombinant Cladophialophora carrionii and Predominantly Clonal Fonsecaea Species
Source: PLoS Negl Trop Dis. 2015 Oct 23;9(10):e0004004. doi: 10.1371/journal.pntd.0004004 (PMC4619687; doi:10.1371/journal.pntd.0004004)
Supplement: S3 Table — (PDF) [file pntd.0004004.s008.pdf]

S3 Table Summary of Max χ^2, GARD and PHI based on the ITS and *BT2* data in *Cladophialophora carrionii* and *Fonsecaea* spp.

**Group**	**Max *chi*^{2}**		**GARD**		**PHI**	
	ITS	*BT2*	ITS	*BT2*	ITS	*BT2*
C.carrionii	13.71 (p = 0.03)	17.75 (p = 0.02)	-	+	P = 0.30	P = 0.02
Fonsecaea spp	25.62 (p = 0.00)	9.32 (p = 0.46)	-	-	P = 0.82	P = 0.21

GARD: genetic analysis for recombination detection
PHI: the pairwise homoplasy index SplitsTree 4
