# Supplementary material for: Global Spread of Human Chromoblastomycosis Is Driven by Recombinant Cladophialophora carrionii and Predominantly Clonal Fonsecaea Species
Source: PLoS Negl Trop Dis. 2015 Oct 23;9(10):e0004004. doi: 10.1371/journal.pntd.0004004 (PMC4619687; doi:10.1371/journal.pntd.0004004)
Supplement: S1 Text — (DOC) [file pntd.0004004.s009.doc]

**S1 Text: The list of strains and cited number presented in the fig.4**

Strains and its number presented in the Fig.4 split decomposition of *Cladophialophora Carrionii* using the concatenated ITS-*BT2* dataset

CBS117901_Venezuela (46) , CBS131844_Japan (42), CBS114394_Venezuela (11), CBS117889_Venezuela (6), CBS117898_Venezuela (66), CBS114395_Venezuela (64), CBS131840_Japan (63), dH14494_unknown (62), CBS114393_Venezuela (61), CBS117895_Venezuela (60), CBS114397_Venezuela (57), CBS410.96_unknown (58), CBS857.96_Venezuela (68), CBS117905_Venezuela (69), CBS131857_Africa (55), CBS117908_Venezuela (14), CBS114403_Venezuela (12), IFM41650_Japan (15), CBS117903_Venezuela (17), CBS131843_China (20), CBS132096_China (21), CBS861.96_Venezuela (22), CBS114402_Venezuela (23), CBS131841_Japan (24), CBS114398_Venezuela (25), CBS108.97_Venezuela (26), CBS131842_Japan (27), CBS131835_China (28), CBS131850_China (30), CBS166.54_Venezuela (38), CBS131846_Japan (39), CBS117909_Venezuela (40), CBS117899_Venezuela (45), CBS131833_Japan (48), CBS117892_Venezuela (49), CBS16454_Venezuela (50), CBS117893_Venezuela (51), CBS117900_Venezuela (52), CBS117902_Venezuela (33),

CBS114396_Venezuela (4), CBS114392_Venezuela (3), CBS114404_Venezuela (53), CBS131851_China (37),

CBS131853_Africa (70), CBS163.54_Australia (29), CBS863.96_Venezuela (54), CBS260.83_Uganda (56),

CBS406.96_Australia (44), CBS131855_Africa (43), CBS109.97_Venezuela (34), CBS162.54_Australia (32),

CBS161.54_Australia (9), CBS100434_Madagascar (10), CBS131839_Japan (47), CBS858.96_Venezuela (65)

CBS114401_Venezuela (19), dH14475_unknown (1), CBS117896_Venezuela (59), CBS859.96_Venezuela (35), CBS117904_Venezuela (36), CBS123392_Mexico (13), CBS131836_Japan (2), CBS114400_Venezuela (5), CBS862.96_Venezuela (31), CBS362.70_Venezuela (16)

IFM41638_Japan (67), IFM41643_Japan (41), CBS114399_Venezuela (8), CBS131854_Africa (18), CBS131856_Africa (7)
